# Covalent modification of primers improves PCR amplification specificity and yield

**DOI:** 10.1093/biomethods/bpx011

**Published:** 2017-11-21

**Authors:** Nancy J Schoenbrunner, Amar P Gupta, Karen K Y Young, Stephen G Will

**Affiliations:** 1 Chemistry and Innovation Technology Department; 2 Department of Infectious Diseases;; 3 Research Department, Roche Molecular Systems, Inc., 4300 Hacienda Drive, Pleasanton, CA 94588, USA

**Keywords:** PCR, primers, modified bases

## Abstract

We report a method for covalent modification of primers that enhances the specificity of PCR and increases the yield of specific amplification products at the end of PCR. The introduction of thermally stable covalent modifications, such as alkyl groups to the exocyclic amines of deoxyadenosine or cytosine residues at the 3′-ends of primers results in enhanced specificity of reactions. This higher specificity can result in greater sensitivity of detection by reducing competition with non-productive reactions. The reduction in the amplification of unintended byproducts is most apparent when both primers are modified at their respective 3′-ends. The *T*_M_s of such modified primers are only slightly affected by the inclusion of these modifiers. The principal mode of action is believed to be driven by the poor enzyme extension of substrates with closely juxtaposed bulky alkyl groups, such as would result from the replication of primer dimer artifact.

## Introduction

The broad application of the PCR process in many areas of molecular biology and to in vitro diagnostic testing is a powerful testament to the specificity and sensitivity of the method. However, there are circumstances where the specificity of the PCR process is compromised through the generation of non-specific artifacts, including primer dimer and other forms of non-specific amplification products, which may limit the ability to sensitively amplify and detect specific sequences. These circumstances include high concentration of total nucleic acids, high concentrations of oligonucleotides, low thermal stringency and partial homology of the primers to non-target sequences. This represents a significant challenge to design sensitive and specific in vitro diagnostic PCR tests for samples composed of complex biological matrices.

One of the key factors determining the specificity of primer-based amplification reactions is the specificity of primer hybridization. Under the elevated temperatures used in a typical amplification reaction, primers hybridize predominantly to their intended target sequences. However, amplification reaction mixtures typically are assembled at room temperature, well below the temperature needed to ensure primer hybridization specificity. The initiation of non-specific amplification is believed to occur during low temperature PCR setup, where there is low but sufficient polymerase activity to extend a primer which is bound weakly or transiently to an unintended, partially complementary templating sequence. This templating sequence could be from the biological sample, e.g. human DNA or from the PCR oligonucleotides themselves. The non-specific amplification can compete for amplification resources, such as primers, dNTPs or the polymerase enzyme. This competition may ultimately limit the efficiency and sensitivity of the amplification and detection of a desired product, especially where a very low copy target is to be detected from a complex biological milieu.

It was recognized early in the development of the PCR process that unintended primer extension products can be produced prior to the formal start of the reaction and these could sometimes compromise the detection of intended target species [1]. Sequencing of primer dimers [2] reveals the presence of two primer sequences (one direct and the other inverted) frequently connected through a short intervening sequence. This intervening sequence may have homology with known sequences, such as from the human genome, or from an included oligonucleotide, but in some cases there may be no identifiable source for these intervening bases.

Clearly, careful primer design can be achieved with advanced software and bioinformatics tools to limit off-target interactions of the primers and improve specificity [3, 4]. However, when working with a clinical target one may be limited in primer design options due to sequence constraints such as limited regions of sequence conservation among the target organisms or sequence similarities to undesired targets such as homologous genes or related organisms. Therefore, additional methods are necessary to improve PCR specificity, and hence the sensitivity of target detection.

Since the inception of PCR, many methods to improve PCR specificity through the reduction of non-specific amplification have been developed [1, 2, 5–25]. Although the DNA polymerase enzymes used in PCR are thermoactive, they still retain some residual activity at lower temperatures [26] and there is some level of primer extension at temperatures lower than those used in the thermocycling profile, such as during PCR assembly. These primer extension reactions can result from hybridization of the primer to non-template sequences under conditions of low hybridization stringency. This is particularly problematic in reverse transcription (RT)-PCR where extended low temperature conditions are often used to reverse transcribe the RNA target.

The term “hot start” is used to indicate methods in which the primer extension rate is dramatically reduced at such lower temperatures by sequestering or altering key reaction components. Upon raising reaction temperatures to those which support the PCR, these restrictions on primer extension rate are removed. However, these methods are technically demanding and not generally suitable to routine diagnostics. Some commonly used hot start PCR techniques reduce enzyme activity at ambient temperature. This has been achieved through the use of antibodies [11, 27] or aptamers [28] that bind specifically to the DNA polymerase. As the temperature is raised during the initiation of the PCR, the antibody or aptamer dissociates from the enzyme, restoring its full activity. Labile, covalent modification of the polymerase enzymes has also been used widely in the field to improve PCR specificity [8, 29]. Reaction of the enzyme with carboxylic anhydrides, such as citraconic or *cis*-aconitic anhydrides, reduces the extension activity of the enzyme. The carboxylic acid residue derived from the anhydride is removed by acid-catalyzed hydrolysis at elevated temperature during an activation phase before normal thermocycling is initiated. In this way, the enzyme is substantially inactive during the low temperature PCR set up phase but its activity is restored on heating. Analogous inactivation schemes have been developed for other PCR components, such as primers [30, 31] and nucleotide triphosphates [32, 33] where the covalent modification can be thermally reversed during a high temperature incubation step.

An alternative reversible inhibition approach has been described in which UV light is used to remove 3′-nitrobenzyl groups from primers in fully assembled PCRs at the elevated temperatures required for specific PCR [24, 25]. These nitrobenzyl groups interfere with the hybridization of the primers to any nucleic acid, thus preventing mismatch extension as would be possible when the primers are annealed to non-intended sequences. This light triggered mode of reversal of modification has the advantage of thermal stability of the nitrobenzylated primers, although it would require the adaptation of standard thermocycling apparatus to allow the illumination of the PCR tubes with UV light to initiate the process.

The principle of oligonucleotide secondary structure has been employed in primer-based hot start techniques. For example, hairpin structures, created by the addition of a six base extension at the 5′-end of a primer sequence that is complementary to the six bases of the 3′-end of the same primer sequence, have been used to prevent hybridization of the 3′-end of the primer until this secondary structure is relieved by melting of the hairpin [20]. Another use of secondary structure to minimize the synthesis of primer dimers has been described [2] whereby the introduction of complementary 5′-tails to primers leads to the formation of a panhandle structure of a primer dimer, which then cannot be propagated.

The use of hot start PCR, whether by labile chemical modification of essential components or by the introduction of thermodynamically moderately stable structures, has clear advantages over standard PCR. However, all of these methods are limited in that they provide only transient inhibition of the initiation of non-specific amplification. Once non-specific amplification has been initiated, amplification of non-specific products can still occur during thermal cycling since these thermally activated primers or analogous components are fully active and can still bind to non-target sequences to some extent and so can still serve to initiate the synthesis of unintended PCR products. The intrinsic instability of these modifications also presents challenges to their use in routine diagnostics. Although reasonable steps can be taken to maintain the stability of the blocking group, there will always be some level of decomposition of the modifying group on storage which will limit the potential beneficial effect over time. Stringent stability control of some of these modified components has been described, including storage at extremely low temperature and the use of non-aqueous solvents [34]. An additional disadvantage of hot start methods that required extended thermal activation is that they are incompatible with some desirable lower temperature processes, such as single-tube, single buffer RT-PCR which uses a 60 °C temperature for the RT step [35].

Some chemically stable modifications of primer sequences have also been reported to improve the specificity of PCRs. For example, 2′-*O*-methyl ribonucleotides at the 3′-ends of primers have been reported to enhance PCR specificity [21]. Similarly, the use of locked nucleic acid residues at the 3′-ends of primers has also been shown to improve detectability of some target sequences [36]. Alkyl groups at the C4′-position of the deoxyribose sugars of nucleotides at the 3′-end of primers have been reported to significantly improve detection of minority variants in allele-specific PCR [37, 38]. Such covalent modifiers can influence the efficiency and stringency of the PCR process in several ways. They can interfere with the thermodynamic stability of the duplex DNA or reduce the ability of the polymerase to extend such primers when bound in weakly hybridized structures, which are postulated in the formation of non-specific amplification products.

The recent description of cooperative primers [39], in which a short priming sequence is covalently linked via non-nucleosidic spacers to a more stable capture sequence, represents an elegant way to suppress the amplification of any primer dimer from such primers. The capture region is synthesized chemically in the reverse (5′–3′) orientation using inverted bases. The linking region serves to allow cooperative binding of the two domains of the primer and to limit the length of the resultant amplicons. The short priming region of the cooperative primer should not bind to a primer dimer and be extended without the additional stabilization afforded by the capture sequence. The authors suggest that once a primer dimer is formed from normal primers, the amplification of this structure proceeds with high efficiency since this dimer is a perfect substrate for further amplification. They also state that these cooperative primers may not decrease the initiation of non-specific amplification, but have their principal benefit in reducing propagation of primer dimer.

**Figure 1:**
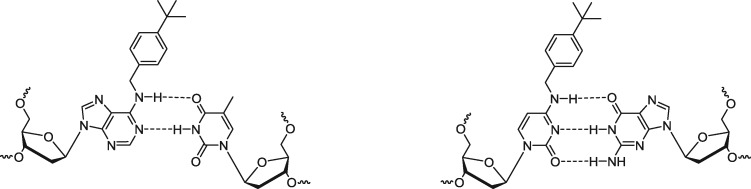
structure of *N*^6^-*p*-tert-butylbenzyl-deoxyadenosine base paired with thymidine and *N*^4^-*p*-tert-butylbenzyl-deoxycytidine base paired with guanosine.

We have devised an alternate stable, covalent modification of primers that results in a reduction of non-specific amplification, especially primer dimer propagation, with concomitant increase in the sensitivity of detection of the intended target amplicon. These primers are modified by covalent attachment of alkyl groups to the exocyclic amines of the adenine or cytidine bases at the 3′-terminal or proximal upstream nucleotides (Fig. 1). Unlike photolabile or thermolabile primer modifications that have been described to date, these stable alkyl modified primers improve overall amplification efficiency by enhancing PCR specificity at every PCR cycle. They serve to interfere with the propagation of primer dimer, since the primer dimers from these modified primers are imperfect substrates for amplification. This will be described in more detail below. Here, we describe the synthesis of the modified primers and their use in PCR amplifications.

## Materials and methods

### Synthesis of alkyl modified primers

The synthesis of modified nucleotides and their corresponding phosphoramidites and controlled pore glass (CPG) substrates is described in the Supplementary Materials.

Modified oligonucleotides were synthesized in three configurations: modifications at the 3′-end only, internal only or doubly modified (3′-end and penultimate). Primers modified at the 3′-terminal base were synthesized using *N*^6^-alkyl-deoxyadenosine or *N*^4^-alkyl-deoxycytidine-modified CPG to initiate the DNA synthesis. Primers modified at an internal base were synthesized using either an *N*^6^-*p*-*tert*-butylbenzyldeoxyadenosine or *N*^4^-ethyldeoxycytidine phosphoramidite. Doubly modified primers were prepared with commercially available *N*^4^-ethyl-dC phosphoramidite (Glen Research, Sterling, VA, USA) in the penultimate positions.

### PCR amplification

The effect of alkyl modification of primers on RT-PCR specificity and sensitivity was examined by amplification of HIV-1 Group O RNA derived from *in vitro* transcription of a cloned plasmid in a pUC-derived vector that included part of the gag gene of the HIV genome [40]. The 20 µl reactions contained 10–10^6^ copies of template RNA in 50 mM tricine, pH 8.3, 90 mM KOAc, 2.5 mM Mn(OAc)_2_, 500 nM each primer, 300 μM dATP, 300 μM dCTP, 300 μM dGTP, 500 μM dUTP, 15% (v/v) glycerol, 1:50,000 dilution of SYBR^®^ Green (Life Technologies, Carlsbad, CA), 20 units Thermus species Z05 DNA polymerase [40] and 2 units AmpErase (Life Technologies).

The primers (Table 1) were either both unmodified (Condition A), both modified with a *p*-*tert*-butylbenzyl (tBuBn) base at the 3′-end (Condition B), or both doubly modified with a 3′-tBuBn group and an additional penultimate *N*^4^-ethyl-dC (Et-dC) residue (Condition C).

**Table 1:** primers used to amplify HIV-1 *gag* RNA

Condition	Forward primer	Reverse primer
A	AATACTATGCTAAATGCCATAGGAGGACA	TGCTAGTTGTTCCAGCAATGTCACTTCC
B	AATACTATGCTAAATGCCATAGGAGGAC**Z**	TGCTAGTTGTTCCAGCAATGTCACTTC**V**
C	AATACTATGCTAAATGCCATAGGAGGA**EZ**	TGCTAGTTGTTCCAGCAATGTCACTT**EV**

Note: E = *N*^4^-Ethyl-dC, Z = *p*-*tert*-butylbenzyl-dA, V = *p*-*tert*-butylbenzyl-dC.

The PCR conditions were 50 °C for 2 min, then 60 °C for 30 min, followed by 50 cycles of 95 °C for 10 s, 60 °C for 30 s using a Light Cycler^®^ 480 DNA real-time PCR system (Roche Applied Science, Mannheim, Germany). The reaction was monitored in real time by SYBR^®^ Green fluorescence. Amplification products were analyzed by electrophoresis through a 2% Nusieve^®^, 0.5% agarose gel followed by ethidium bromide staining.

The effect of alkyl modification of primers was further tested by amplifying *Mycobacterium tuberculosis* DNA diluted into either buffer or processed sputum samples. DNA was purified from cultured *M. tuberculosis* and was diluted to 10 copies/µl in either buffer (a 1:1 mixture of lysis and neutralization reagents from the Amplicor™ MTB Test kit; Roche Molecular Systems, Pleasanton, CA, USA) or in pooled crude lysate from liquefied sputum sediments.

Decontaminated and liquefied sputum sediments, previously shown to be negative for mycobacteria by microscopy and culture (purchased from San Francisco General Hospital), were processed as previously described [41]. Briefly, 100 μl of sputum sediment were added to 500 μl of Respiratory Specimen Wash Reagent and centrifuged for 10 min at 12 500 G. The resultant pellet was resuspended in 100 µl of lysis reagent and incubated for 45 min at 60 °C. The lysate was then neutralized with 100 μl of neutralization reagent. Eight different negative sputum lysate pools, each composed of two individual sputum lysates, were created to mimic clinical background.

Amplifications were carried out in 100 μl reactions containing 10 copies of template DNA diluted in either buffer or sputum lysate; 50 mM Tris–HCl, pH 8.9; 250 nM each primer; 100 μM each of dATP, dCTP, dGTP and dUTP; 10% (v/v) glycerol; 5 units AmpliTaq^®^ (Life Technologies) and 2 units AmpErase^®^ (Life Technologies). The primers were derived from a region of the 16 S rRNA gene that is conserved among mycobacterial species (Table 2) [41]. The primers were either unmodified or were modified by the inclusion of benzyl groups at the 3′-end.

**Table 2:** primers used to amplify *M. tuberculosis* DNA

Condition	Forward primer	Reverse primer
A	CACATGCAAGTCGAACGGAAAGG	GCCCGTATCGCCCGCACGCTCACA
B	TAACACATGCAAGTCGAACGGAAA	GCCCGTATCGCCCGCACGCTCACA
C	TAACACATGCAAGTCGAACGGAA**K**	GCCCGTATCGCCCGCACGCTCAC**K**

Note: K = *N*^6^-benzyl-dA.

The PCR conditions were 50 °C for 5 min, followed by 2 cycles of 98 °C for 20 s, 62 °C for 20 s, 72 °C for 45 s, followed by 41 cycles of 94 °C for 20 s, 62 °C for 20 s, 72 °C for 45 s and a final incubation at 72°C for >10 min using an Applied Biosystems 9600 DNA Thermal cycler (Life Technologies). Amplification products were analyzed by electrophoresis through a 2% Nusieve^®^, 0.5% agarose gel followed by ethidium bromide staining.

### Kinetics of primer extension

To determine how alkylation of the exocyclic amines of the DNA substrate affected enzyme activity, the time course of primer extension reactions was monitored. Ternary complexes of *Taq* polymerase and models of primer template hybrids were prepared and extension kinetics measured under static quenched flow conditions. Two oligonucleotides were hybridized to create a model of a PCR primer and its corresponding template by annealing a 1.2-fold molar excess of template over primer. The primer sequence was labeled at its 5′-end with 6-carboxyfluorescein (FAM) for subsequent detection by electrophoretic separation (5′-FAM-CCCTCGCAGCCGTCCAACCAACTCA-3′) and the template (3′-GGGAGCGTCGGCAGGTTGGTTGAGTAGGTCTTGTTT-5′) were created to position the two tBuBn-modified bases in close proximity; underlined bases indicate positions modified with tBuBn moieties. The primer and template oligonucleotides each potentially carried one alkylated dA, at the 3′-end of the primer or at the base opposite the first incoming TTP in the template. All four permutations of modified and unmodified primer–template duplexes were used as substrates in single nucleotide addition reactions. These reactions were carried out at 50 °C with 2.5 µM annealed primer-template and 100 nM *Taq* DNA Polymerase (Roche Applied Sciences, Penzberg, Germany) in 10 mM Tris, pH 8.3, 50 mM KCl, 100 µM TTP, 2 mM MgCl_2_ and were quenched after 10–40 s by the addition of EDTA (50 mM final). The fluorescent extension products were separated and detected by capillary electrophoresis (ABI PRISM^®^ 3100; Life Technologies). The relative amounts of the full-length and extended oligonucleotides were quantitated by GENESCAN Software (Life Technologies). The steady-state rate (*k*_2_), the burst amplitude (*A*, which is equal to the concentration of polymerase active site) and the initial rate of product formation (*r*, the burst rate) were extrapolated from the burst equation [42].

(1) $$\left[\text{product}\right]=A\left(1-e^{-rt}\right)+k_{2}t.$$

In some cases the first time point was in the steady-state phase ((1−e^−^^*rt*^) ∼ 1), and so only *A* and *k*_2_ could be determined.

### Molecular modeling

Starting with the structure of the *Taq* DNA polymerase ternary complex with primer-template and dNTP (3ktq.pdb) [43] the base pairs of the primer terminus and the incoming nucleotide were changed from dC-dG to dA-dT. *Tert*-butylbenzyl groups were model built on the *N*^6^-group of the 3′-terminal dA of the primer and the dA across from incoming TTP in the template strand using the program Moloc [44]. After model building, the structures were subjected to restrained energy minimization.

### *T*_M_ determination

Oligonucleotide thermal melting experiments were performed on a LightCycler^®^ 480 (Roche Applied Sciences) instrument. Complementary oligonucleotide pairs as shown in Table 4 were combined at 0.25 µM or 2.5 µM each in a buffer containing 10 mM Tris (pH 8.3), 50 mM KCl, 100 µM TTP, 2 mM MgCl_2_ and 10 nM SYTO^®^ 13 dye (Life Technologies). The temperature was raised from 40°C to 90°C over 25 min and the fluorescence (excitation 465 nm, emission 510 nm) was monitored continuously. The decrease in fluorescence resulting from strand separation resulted in a characteristic melting curve and the *T*_m_ values were obtained from the first derivatives of the fluorescence curves.

## Results

### HIV-1 RNA amplifications using benzyl-modified primers

**Figure 2:**
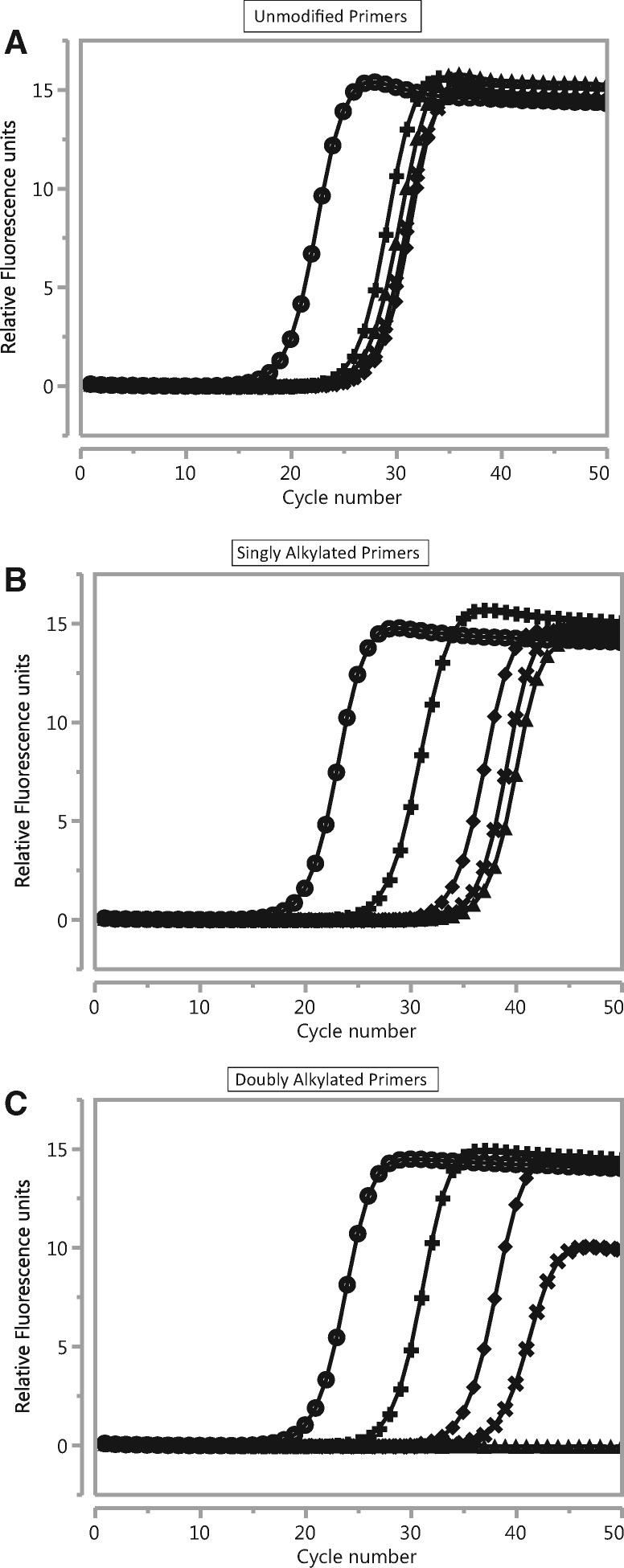
Real-time RT-PCR amplification plots of HIV Group O RNA at 10^6^ (open circle), 10^4^ (cross), 10^2^ (filled diamond), 10 (X) and 0 (NTC, no template control, filled triangle) copy starting RNA templates per reaction, as monitored by SYBR Green® fluorescence for (**A**) unmodified, (**B**) singly alkylated or (**C**) doubly alkylated primer pairs.

**Figure 3:**
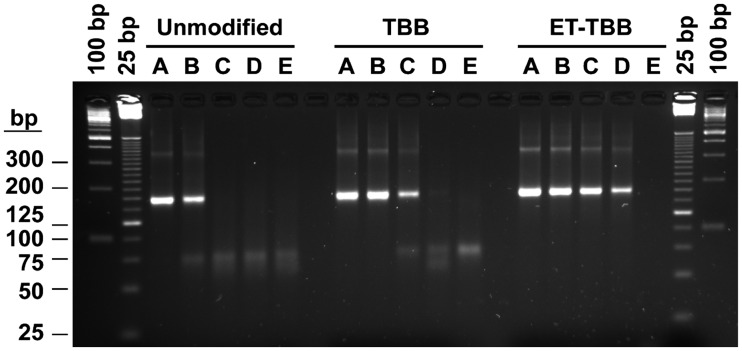
RT-PCR products of HIV1 Group O amplification with (**A**) 10^6^, (**B**) 10^4^, (**C**) 10^2^, (**D**) 10 and (**E**) 0 copy starting template per reaction; gel analysis. Lanes marked 100 and 25 bp contain MW ladders with respective size spacing. Unmodified refers to the control primer set with both primers unmodified; TBB refers to the condition where the primer pair was modified with a 3′-tBuBn-dA or -dC moiety; ET-TBB refers to the condition with doubly modified primers, with an N^4^-ethyl-dC modification at the (*n*−1) position in addition to the 3′-terminal tBuBn deoxyadenosine or deoxycytosine moiety.

An HIV-1 Group O RNA template was amplified with three different primer sets. The nucleotide sequences of the primer sets were identical and varied only in the nature and degree of chemical modification of the bases at the 3′-terminus (Table 1). In the control primer set, both primers were unmodified. A second primer pair was singly modified with a 3′-tBuBn-dA or -dC moiety. The third primer set consisted of doubly modified primers, with an *N*^4^-ethyl-dC modification at the (*n*−1) position in addition to the 3′-terminal tBuBn deoxyadenosine or deoxycytosine moiety. Amplification of a range of input target (10^6^–10 copies HIV RNA per PCR) was monitored by real-time detection using SYBR^®^ Green [45] (Fig. 2) and the resulting amplification products were analyzed by gel electrophoresis (Fig. 3).

Real-time growth curves for the control primer set showed earlier *C*_t_ values for 10^6^ and 10^4^ copy input than for either of the two modified primer sets (Fig. 2). However, the growth curves for the 10^2^ copy, 10 copy and the No Template Control (NTC) reactions were indistinguishable from one another (Fig. 2A). Gel analysis of the PCR products from these reactions (Fig. 3) only showed the expected amplicon for reactions with 10^6^ and 10^4^ copy input. At lower or at zero input copy levels, a low molecular weight band consistent with primer dimer artifact was observed. This indicates that non-specific amplification predominates below 10^4^ copy target input in these reactions with unmodified primers.

For the singly modified primer set, the 10^2^ copy input reaction is now distinguishable from the NTC, but the 10 copy reaction is not (Fig. 2B). Gel analysis correlates with the real-time detection: a specific product band is observed for reactions down to 10^2^ copy input, while only primer dimer is observed at 10 copy and NTC reactions (Fig. 3). For the doubly alkylated primer set, none of the NTC reactions yield a growth curve and 2 out of 3 of the 10 copy reactions yield a growth curve (Fig. 2C). Again, the gel analysis of the doubly alkylated primer PCRs correlates with the real-time detection results, with specific amplicon evident for two of the three 10-copy reactions and no primer dimer in the NTCs (Fig. 3). Specific amplicon yield, based on staining intensity of the expected PCR product on the gel, increases with the increasing level of alkylation, despite the delayed *C*_t_’s of growth curves with modified primers. This improvement in PCR yield is most significant for low copy target inputs.

### PCR amplification of *M.tuberculosis* DNA

**Figure 4:**
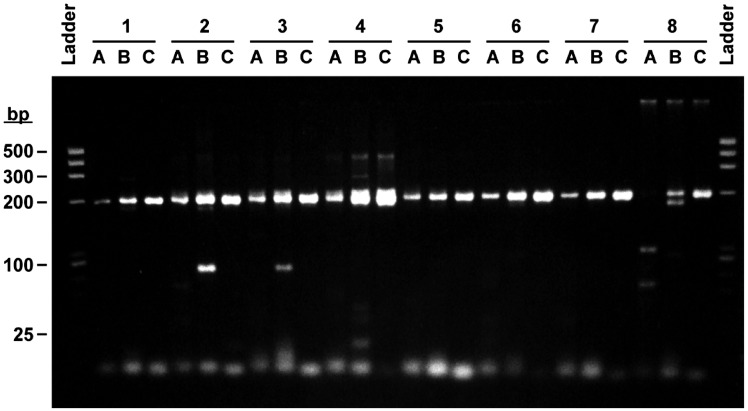
amplification of *M. tuberculosis* DNA spiked into eight different processed pooled negative sputum lysate. The amplifications were performed with either unmodified primers (**A** and **B**) or alkylated primers (**C**) as shown in Table 2. The intended mycobacterial amplicon is the major 200 bp product seen in the gels. Other bands correspond to non-specific amplification product; the lowest bands in the gel correspond to primer dimer. Lanes marked as “Ladder” contain a molecular weight marker (Hae III digestion of PhiX174 DNA).

In order to understand the effect of primer alkylation on PCR in a clinical matrix, the amplification of *M. tuberculosis DNA* in eight unique crude sputum lysates was analyzed. The results of the electrophoretic analysis are shown in Fig. 4. Two sets of unmodified primers (A and B) and one set of modified primers (C) were compared (Table 2). The upstream primer of set A contains a nucleotide at its 3′-terminus which cannot be modified. Primer set B shares a downstream primer with set A, but the upstream primer was shifted slightly such that the 3′-terminal nucleotide is one that can be alkyl-modified. The primers of set C are alkyl-modified versions of the primers in set B. Different amounts of specific and non-specific products, including primer dimer, were observed in each of the primer combinations. For the amplifications conducted in the eight clinical sputum matrices, generally more non-specific products and primer dimer, and less specific product was observed for the unmodified primer sets A and B than for the alkylated primer set C. The reactions using primer set C were extremely clean with no unintended side products and with a higher yield of the specific amplicon.

### Kinetics

**Figure 5:**
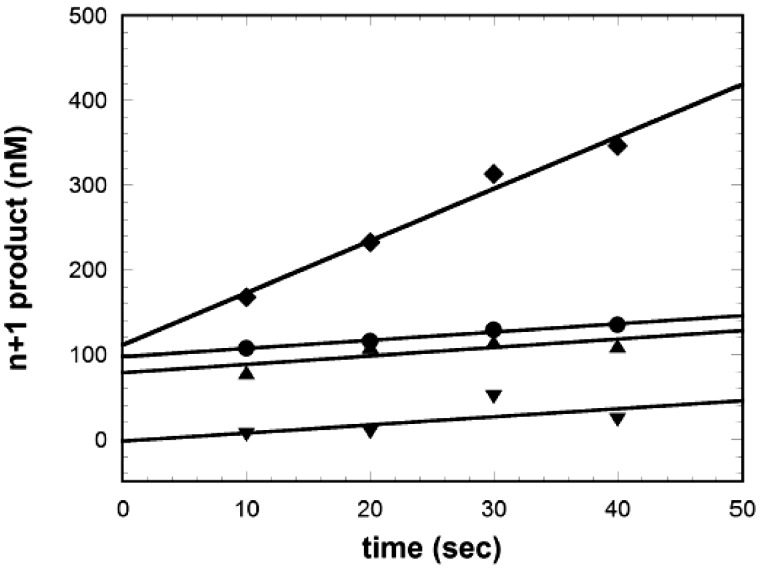
single nucleotide addition kinetics with oligonucleotide primer-template substrate with no modifications (filled circles), single alkyl group at the 3′-end of the primer (filled triangles), single alkyl group at the templating base (filled diamonds) or doubly alkylated at both the 3′-end of the primer and at the templating base (filled inverted triangles).

In order to elucidate the mechanism by which the alkyl modifications affect the specificity of PCR, the steady-state kinetics of single-nucleotide addition by *Taq* polymerase to various primer–template substrates (Fig. 5) were measured. With unmodified primer–template duplexes (NJS01–NJS03), the expected burst kinetics were observed: there is a rapid formation of single-nucleotide extension product in the pre-steady-state phase within the dead time of the experiment (10 s), followed by the slow, steady-state formation of additional single-nucleotide extension products. The amplitude of the burst phase corresponds to the concentration of active enzyme. The steady-state phase reflects the slower rate of additional product turnover limited by the rate of enzyme cycling, i.e. for the enzyme to dissociate from the complex after nucleotide extension and bind to another non-extended complex to repeat nucleotide incorporation [35]. The burst amplitude and steady-state rate constants determined by fitting the data to Equation (1) are shown in Table 3.

**Table 3:** kinetic parameters for steady-state single-nucleotide addition

Substrate (primer-template)	Burst kinetics?	Burst amplitude (nM)	Kss (s^−1^)
Unmodified primer and template	Yes	97.6	0.01
tBuBn-primer	Yes	78.6	0.01
tBuBn-template	Yes	111.1	0.06
tBuBn-primer, tBuBn-template	No	−2.0	0.01

Note: unmod = unmodified primer-template; tBuBn-primer = 3′-tBuBn-dA-modified primer/unmodified template; tBuBn-dA at templating base; double tBuBn = 3′-tBuBn-dA-modified primer/tBuBn-dA at templating base.

The 3′-tBuBn-dA primer annealed to unmodified template represents the condition of the initial extension of modified primer annealed to target. The time course with this substrate is essentially identical to that with unmodified primer–template: there is a pre-steady-state burst phase of identical amplitude, followed by a steady-state phase with comparable kinetics.

The effect of a tBuBn group at the templating base, which mimics the situation of replicating across from a modified primer extended in the previous cycle of the PCR, can additionally be seen in Fig. 5. The time course of single-nucleotide addition to this substrate also displays a burst phase with amplitude equivalent to the concentration of active *Taq* enzyme. The steady-state phase, however, is six-fold faster than with the unmodified primer–template, indicating that the enzyme dissociates significantly faster from this substrate following primer extension. In other words, the tBuBn modification in the template position interferes with the enzyme binding to the substrate.

Finally, the effect of having a tBuBn group at both priming and templating bases, which mimics propagation of a previously initiated primer dimer formed from two 3′-terminally modified primers, is the lowest curve seen in Fig. 5. The rates of single-nucleotide kinetics on this substrate are dramatically different. No burst phase is observed. This implies that the observed steady-state kinetic actually represents the first round of nucleotide addition at the very slow rate of 0.01 s^−1^, which is at least 50 times slower than unmodified or singly alkylated substrates (Table 3).

### Molecular modeling

**Figure 6:**
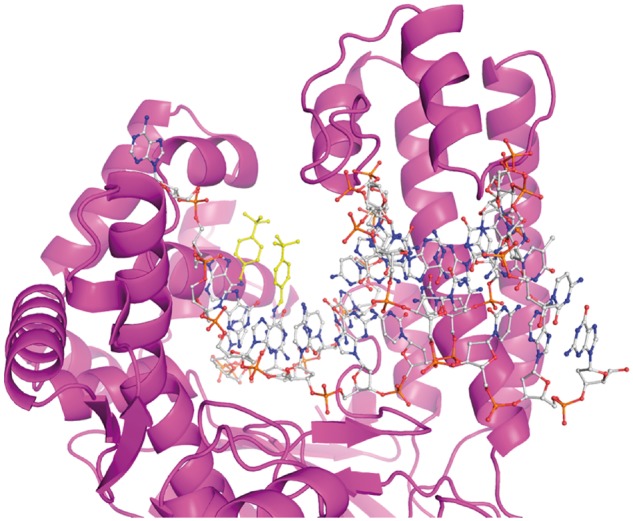
homology modeled structure of Taq DNA polymerase in a ternary complex with primer-template, dNTP and divalent metal cofactor. Both the 3′-end of the primer and the templating base are modified with a *N*^6^-*tert*-butylbenzyl-deoxyadenosine.

*Taq* DNA polymerase is highly homologous to Thermus species Z05 (91% identity) (T. Myers, personal communication). The crystal structure of Taq DNA polymerase [43] bound to a double-stranded DNA substrate was used to model build an alkylated primer–template. This model provides a structural context to help understand the effects of these primer modifications during the various scenarios encountered during PCR. Figure 6 shows a model of *Taq* DNA polymerase bound to a double-stranded DNA substrate with both the bases at the 3′-end of the primer and the templating base opposite the incoming dNTP modified with tert-butylbenzyl groups. Such a doubly alkylated DNA would occur during the replication of a previously initiated primer dimer, although not if there were extensive intervening sequence between the primers. The two bulky alkyl moieties are closely juxtaposed to one another in this structure. Such doubly modified DNA with a high degree of steric interference between the two bulky adducts and between the modifiers and the enzyme would clearly impair catalytic activity. On the other hand, singly alkylated structures, with either an alkylated priming or templating base, would be found upon primer annealing or upon replication of a primer extension product in subsequent cycles. These individual *tert*-butylbenzyl groups point into the large cleft of the enzyme on the major groove face of the double-stranded DNA. Thus, a single tBuBn group, either on the priming or on the templating strand, is structurally well tolerated.

### Thermal melting

To evaluate the effect of the benzyl modifications on the melting temperature of a primer, several oligonucleotides were prepared with or without a tBuBn modification. In addition to evaluating the effect of the modification on the 3′-terminally modified primers, the *T*_m_s of internally modified sequences were also compared with the unmodified analogs. The various sequences and the melting results are shown in the Supplementary Materials and Table 4. The thermal transition was monitored fluorescently using SYTO-13, a DNA-binding dye which has been shown not to influence melting temperature of DNA [46], although differential dye effects in the presence of a mismatched base pair cannot be entirely excluded. There was no observable difference in *T*_m_s when a single modification was used at the 3′-terminal dA base. When a tBuBn-modified deoxyadenosine was placed internally within the sequence in either strand, a 3–5 °C reduction in melting temperature was observed. When a modification was present internally in both strands, in such a way that two bulky modifications are at adjacent positions on opposite strands, a *T*_m_ reduction of 7 °C was observed.

**Table 4:** oligonucleotides used were either unmodified or modified with a single *tert*-butylbenzyl-dA substitution indicated as Z within the sequence

Oligonucleotide pair	*T*_m_ (°C)
5’-CCCTCGCAGCCGTCCAACCAACTCA-3’	75
3’-GGGAGCGTCGGCAGGTTGGTTGAGTAGGTCTTGTTT-5’	
5’-CCCTCGCAGCCGTCCAACCAACTC**Z**-**3’**	75
3’-GGGAGCGTCGGCAGGTTGGTTGAGTAGGTCTTGTTT-5’	
5’-TTTGTTCTGGATGAGTTGGTTGGACGGCTGCGAGGG-3’	61
3’-CAAGACCTACTCAACCAA-5’	
5’-TTTGTTCTGGATGAGTTGGTTGGACGGCTGCGAGGG-3’	56
3’-CAAGACCT**Z**CTCAACCAA-5’	
5’-TTTGTTCTGG**Z**TGAGTTGGTTGGACGGCTGCGAGGG-3’	57
3’-CAAGACCTACTCAACCAA-5’	
5’-TTTGTTCTGG**Z**TGAGTTGGTTGGACGGCTGCGAGGG-3’	53
3’-CAAGACCT**Z**CTCAACCAA-5’	

Notes: Z = *tert*-butylbenzyl-dA, E = *N*^4^-Ethyl-dC, M = *N*^6^-Methyl-dA, B = Benzyl dA, V= *tert*-butylbenzyl-dC. *T*_m_ values shown were those determined with oligonucleotide concentration of 0.25 μM.

## Discussion

Although many methods to improve the specificity of PCR have been described, each of these methods involves some compromise in assay design, implementation or performance. We have developed an alternative method for specificity improvement using chemically stable primer modifications as a convenient and broadly applicable solution to this problem. This new method has many advantages over earlier techniques including the facile chemical synthesis of such modified primers using conventional DNA synthesis methods. A modified CPG DNA synthesis substrate is required as the only novel reagent, and the primers are easily produced under normal DNA synthesis and deprotection conditions. They are also stable under standard oligonucleotide storage conditions. These benzyl modified primers have been shown to be compatible with PCR amplification of DNA targets and the single enzyme, single buffer Mn^2+^ catalyzed RT-PCR of RNA targets [47]. This is an attractive attribute of the method since, unlike other methods using thermally activated PCR components, no high temperature activation step is needed which would otherwise risk destruction of labile or rare RNA targets. The retention of the covalent modifier in the primer potentially guards against mis-extension in every cycle of PCR with concomitant specificity enhancement.

Although three of the naturally occurring nucleotide bases in DNA are potentially modifiable by the inclusion of a benzyl group on exocyclic amines, only deoxyadenosine and deoxycytidine are attractive targets for this type of modifier. The benzyl group on the exocyclic amine of these bases is directed toward the DNA major groove, away from the interaction surface of primer–template duplexes bound to DNA polymerases [42, 43]. Hence, the otherwise bulky adducts are likely to be well tolerated in the enzyme active site. In analogously modified deoxyguanosine the benzyl group is directed toward the minor groove which does interact with the protein surface, and so would likely interfere with the normal functioning of the polymerase [48].

We have described here the evaluation of these modified primers in applications to illustrate the diversity of possible modifications that could be potentially introduced into primers. The example of amplification of *M. tuberculosis* DNA makes use of two primers, each with a deoxyadenosine at the 3′-end of its sequence. In our study, each of these residues was modified as *N*^6^-Benzyl dA, and in this case the amplification was carried out in the presence of *Taq* polymerase with Magnesium as enzymatic cofactor. The other example in our study uses a reaction mix including the Z05 polymerase with Manganese for single enzyme RT-PCR with primers modified at their 3′-ends with *tert*-butyl benzyl groups. This example also makes use of the ability to modify the exocyclic amine of deoxycytidine for one of the primers in combination with the other primer modified at the 3′-deoxyadenosine. The 3′-dC primer also includes an additional, less bulky exocyclic amine modifier, *N*^4^-Methyl dC, at an adjacent nucleotide position. The specificity enhancement of the primer modifications is directly correlated with the size of the hydrophobic group and its proximity to the 3′-end. However, excessive primer modification with respect to size and number can lead to decreased ability to extend a primer, and hence negatively impact sensitivity. The variety of type and location of primer modifications allows an appropriate balance to be struck between specificity and sensitivity.

The HIV RNA amplification results above illustrate how the detection of low level viral RNA can be compromised by non-specific amplification when unmodified primers are used. The introduction of benzylated primers offers a clear advantage in this situation. While there may be a slight delay in *C*_t_ values, the production of primer dimer and other non-specific side products is significantly reduced. The delayed *C*_t_ values may possibly be corrected by extending the annealing/extension time of the assay. Ultimately, even with *C*_t_ delays, the yield of the desired product is dramatically increased with alkyl-modified primers, allowing the detection of very few molecules of target. This translates into greater sensitivity.

The PCR detection of M. tuberculosis DNA from liquefied sputum poses practical challenges due to the inherent complexity of this sample type. Non-specific amplification, facilitated by factors inherent in the sample matrix, such as the high burden of DNA from lysed human and bacterial cells in the samples, can compromise the ability to amplify and detect specific DNA sequences from target organisms. The use of benzylated primers has been shown to be extremely beneficial for the detection of M. tuberculosis. Gel analysis (Fig. 4) shows significant reduction of non-specific amplification products and corresponding increase of specific products generated with these primers. These data also illustrate that the production of non-specific amplification products other than primer dimers can also be suppressed through the use of these modified nucleotides. The application of these modified primers for the detection of low-copy clinical targets, in either model samples (HIV) or from a complex clinical background (M. tuberculosis), demonstrates the potential benefit of these modifications to the detection of clinically relevant targets and samples. The experimental examination of what takes place during initial PCR cycles is challenging due to the stochastic nature of the initiation of primer extension arising from non-homologous targets, compounded with the extremely low concentrations of these initial primer extension products. Although the experimental study of such rare events under active thermocycling conditions is exceedingly difficult to perform, we have investigated primer extension kinetics, the thermodynamics of hybridization and structural modeling to suggest the mode of action of these alkylated primers. These physico-chemical properties were examined for two configurations of DNA duplexes; one in which a single benzyl group is present—as would be formed in a normal PCR product of around 100 base pairs long—and one in which two modifiers are in close proximity as would be obtained in a primer dimer structure. A model for the initiation and propagation of non-specific amplification products, and how such side reactions are mitigated by alkylated primers, can be drawn.

Non-specific PCR products, whether primer dimer artifacts or amplification of heterologous nucleic acid in the sample, are likely initiated by annealing and extension of primers bound to partially complementary targets, which is favored at lower temperatures. Primer extension kinetics on such poorly matched duplexes are extremely slow [49], which can be explained by the less than ideal alignment of the primer–template substrate in the DNA polymerase active site. If a primer is further modified with a bulky alkyl group, this steric hindrance would be further exacerbated and the extension kinetics would likely be slowed even further [50], thus reducing the initiation of the non-specific amplification.

From the molecular modeling studies, it can be seen that a single benzyl modification on these primers can be accommodated within the polymerase active site without any significant steric interference and has no conspicuous effect on the steric trajectory of DNA synthesis. These modifications are therefore well tolerated by the polymerase enzymes when the primer is perfectly matched to the template. Although the presence of a benzyl group on the exocyclic amine of a 3′-terminal base of a primer does not prevent Watson–Crick base pairing with its complementary base, there may be a kinetic barrier to the formation of this base pair since only one hydrogen atom capable of hydrogen bonding remains on the exocyclic amine. It may be that this kinetic barrier reduces the efficiency with which a primer, transiently bound to an imperfectly matched sequence, is extended by the polymerase. However, a perfectly matched primer may bind to its complementary sequence for sufficient time to form this 3′-terminal hydrogen bond, and hence may serve as substrate for efficient polymerase extension. There is almost no thermodynamic destabilization of a 3′-benzylated primer when annealed to its complement, which again suggests minimal impact of these modifications on primer hybridization.

To further test the model, a hybridized oligonucleotide pair, mimicking the formation of a simple primer dimer structure derived from primers containing benzylated nucleotides, was also examined. In such a structure, the two benzyl groups are in very close proximity, one at the 3′-end of a priming oligonucleotide, and the second placed internally within a sequence as would be obtained by primer extension in an earlier round of PCR cycling (Fig. 6). In this case, the *T*_m_ is significantly reduced relative to an unmodified primer, indicating a substantial thermodynamic destabilization of the hybrid structure where two benzyl groups are adjacent but on opposite strands. Molecular modeling of this structure shows that such a doubly alkylated primer–template DNA duplex is poorly accommodated in the active site of a polymerase. Indeed, the steady-state enzyme kinetic data show that a primer–template duplex of this type is very poorly extended by the polymerase, suggesting that a severe kinetic barrier exists to the extension of the primer in such a hybrid structure. The propagation of primer dimers in PCR, through exponential amplification, is normally a very efficient process due to the small size of the primer dimers. The presence of alkylated nucleotides at the 3′-ends of both primers may serve to significantly reduce the efficiency of primer dimer propagation in PCR, through a combination of these thermodynamic and kinetic effects. While alkylated primers are tolerated and extended efficiently when annealed to the complementary, intended target, non-specific amplification is suppressed. While this kinetic and steric hindrance model may offer an attractive explanation, it is not consistent with all data; specifically the fact that sequenced primer dimer contains intervening sequence between the primers.

The physico-chemical studies above suggest the potential of alkylated primers to inhibit both the initiation of non-specific amplification, and the initiation and propagation of primer dimer. This can explain the improved specificity of PCR. Although overall PCR efficiency may be slightly impacted by the presence of a benzyl group in the primers, and this impact may delay the *C*_t_ value for a given input of target, the compensating increase in robustness and assay sensitivity outweighs the effect of a marginal *C*_t_ delay. The increased specificity allows additional amplification cycles to be performed.

The applications shown here, sensitive RNA detection and DNA amplification from complex sample matrices, represent some of the greatest challenges in clinical molecular diagnostics. The benzyl primers improve specificity and, thus, sensitivity of PCR in both cases. Although this technology may be particularly useful in amplification reactions where the intended targets are of low abundance, they may also offer advantages when the target of interest is highly homologous to related, non-target, sequences and in those cases where factors favoring non-specific amplification cannot be avoided, such as high oligonucleotide concentration or multiplex PCR.

## Supplementary data

Supplementary data are available at *Biology Methods and Protocols* online.

## Supplementary Material

Supplementary Data
