# Supplementary material for: Covalent modification of primers improves PCR amplification specificity and yield
Source: Biol Methods Protoc. 2017 Nov 21;2(1):bpx011. doi: 10.1093/biomethods/bpx011 (PMC6994073; doi:10.1093/biomethods/bpx011)
Supplement: Supplementary Data [file bpx011_supp.docx]

SUPPLEMENTARY

SYNTHESIS OF HIGH SPECIFICITY PRIMERS CONTAINING MODIFIED BASES

Synthesis of Alkyl Modified Primers. CPG Synthesis.

Modified oligonucleotides were synthesized in three configurations: modifications at the 3’ end only, internal only or doubly modified (3’ end and penultimate). Primers modified at the 3’ terminal base were synthesized using N^6^-alkyl-deoxyadenosine or N^4^-alkyl-deoxycytidine modified Controlled Pore Glass (CPG) to initiate the DNA synthesis. Primers modified at an internal base were synthesized using either an N^6^- p-tert-butylbenzyldeoxyadenosine- or N^4^-ethyldeoxycytidine phosphoramidite.  Doubly modified primers were prepared with commercially available N^4^-Ethyl-dC phosphoramidite (Glen Research, Sterling, VA, USA) in the penultimate positions.

The chemical synthesis of N^6^-benzyldeoxyadenosine CPG, and N^6^-benzyldeoxyadenosine phosphoramidite are described below. The synthesis of the corresponding N^6^- p-tert-butylbenzyldeoxyadenosine analogs was performed in an identical fashion, except p-tertbutylbenzyl bromide replaced benzyl bromide in the first step.

1. Synthesis of N^6^-benzyldeoxyadenosine CPG

Step 1: Synthesis of N^6^-benzoyl, N^6^-benzyl, 5’-O-DMT-2’-deoxyadenosine

To N^6^-Benzoyl-5’-O-(4,4’-dimethoxytrityl)-2’-deoxyadenosine ( 657 mg, 1.0 mmol; Aldrich Chemical Co., Milwaukee, WI), pyridine (10 ml) was added and the mixture was dried by evaporation under vacuum. This was repeated. The resulting foam was dissolved in anhydrous DMF^1^ (15 ml; Aldrich Chemical Co., Milwaukee, WI) and cooled to 5^o^C. Sodium hydride (44 mg, 1.1 mmol, 1.1 equiv. 60% dispersion in oil) was added under an argon atmosphere and stirred at room temperature for 45 minutes. Benzyl bromide (143 μl, 206 mg, 1.2 mmol, 1.2 equiv; Aldrich Chemical Co., Milwaukee, WI) was added over 2 minutes and the mixture was stirred overnight at room temperature. The mixture was dried by evaporation under vacuum and the residue was partitioned between ethyl acetate and water (10 ml each) and extracted. The aqueous phase was re-extracted with ethyl acetate (10 ml) and the combined extracts were dried over anhydrous magnesium sulfate, filtered and evaporated. The crude product was purified by column chromatography on silica gel (75 g) using methanol, triethylamine, methylene chloride (3:0.5:96.5). Fractions containing the product were combined and dried by evaporation to give the expected N^6^-benzoyl, N^6^-benzyl, 5’-O-DMT-2’-deoxyadenosine (410 mg, 54%). The structure of the product was confirmed by NMR.

Step 2: Succinylation

To the N^6^-benzoyl, N^6^-benzyl, 5’-O-DMT-2’-deoxyadenosine (295 mg, 0.39 mmol), pyridine (10 ml) was added and the mixture was dried by evaporation under high vacuum. This step was repeated. Fresh anhydrous pyridine (10 ml) was added together with succinic anhydride (200 mg, 2 mmol, 5.0 equiv) and DMAP (24 mg), and the solution was stirred under an argon atmosphere overnight at room temperature. The bulk of the solvent was removed under vacuum and the residue was partitioned between methylene chloride (20 ml) and sodium citrate solution (20 ml, 0.1 M, pH 5.0) and extracted. The aqueous phase was extracted with more methylene chloride (20 ml) and the combined extracts were dried over anhydrous sodium sulfate, filtered, and dried by evaporation. The product was purified by column chromatography on silica gel (4.5 g) using ethyl acetate, triethylamine, methylene chloride (32:1:67) to give the expected 3’-succinate ester, N^6^-benzoyl-N^6^-benzyl-3’-O-succinate-5’-O-DMT-2’-deoxyadenosine (247 mg, 74%).

Step 3: Derivatization of CPG

Acid washed CPG was prepared a follows. LCAA-CPG (1.0 g, LCA00500C, 500 angstrom, 88.6 μmol/g; CPG Inc., Fairfield, NJ) was washed with dichloroacetic acid in dichloromethane (2%, 20 ml) by swirling periodically over 20 minutes at room temperature. The acid washed CPG was filtered on a glass frit and washed with dichloromethane until acid free. The powder was air dried, then dried under vacuum at room temperature overnight.

Coupling of the modified nucleoside intermediate to the acid washed CPG was carried out as follows. To a solution of N^6^-Benzoyl-N^6^-benzyl-3’-O-succinate-5’-O-DMT-2’-deoxyadenosine (170 mg, 0.2 mmol), prepared as described above, in dichloromethane (10 ml) was added TEA (100 μL), and the solution was concentrated to approximately 5 ml under an argon atmosphere. DMAP (12 mg, 0.1 mmol, 0.5 equiv), TEA (100 μL), EDC (384 mg, 2.0 mmol, 10 equiv), and the acid-washed CPG from above were added in sequence. Anhydrous pyridine (5 ml) was added and the mixture was sealed and shaken for 3 days at room temperature. The CPG was filtered off under vacuum and washed extensively with isopropanol, then with dichloromethane, air dried, then dried under vacuum for 1 hour.

Capping of the derivatized CPG was carried out as follows. To the dry derivatized CPG were added Cap A and Cap B solutions (5 ml each, Acetic anhydride/ 2,6-Lutidine/THF and 10% N-Methylimidazole in THF; Glen Research DNA synthesis reagents, Sterling, VA) and the mixture was shaken for 4 hours at room temperature. The CPG was filtered off under vacuum and washed extensively with isopropanol, then dichloromethane, air dried, then dried under vacuum overnight.

II. Synthesis of N^6^-Benzyl Deoxyadenosine Phosphoramidite.

N^6^-benzoyl, N^6^-benzyl, 5’-O-DMT-2’-deoxyadenosine was synthesized as described above.

To N^6^-benzoyl, N^6^-benzyl, 5’-O-DMT-2’-deoxyadenosine (196 mg, 0.26 mmol) in dry THF (8 ml) was added diisopropylethylamine (350 μL, 270 mg, 2.04 mmol, 7.8 equiv) and 2-cyanoethyl N,N-diisopropylchlorophosphoramidite (161 mg, 0.68 mmol, 2.6 equiv.; Aldrich Chemical Co., Milwaukee, WI), and the mixture was stirred for 30 minutes at room temperature under an argon atmosphere. The solvent was removed under vacuum and the residue was partitioned between sodium bicarbonate solution (5%, 20 ml) and ethyl acetate (20 ml). The organic phase was washed with the bicarbonate solution, water, and saturated brine (20 ml each) in sequence, dried over sodium sulfate, filtered, and evaporated. The residue was purified by column chromatography on silica gel (4 g) using acetone/hexane/TEA (34:65:0.7) to yield the desired phosphoramidite (248 mg, 100%).

DNA Synthesis, purification and analysis of Primers modified with a Benzyl group.

Benzyl and p-tert-butylbenzyl modified primers were synthesized by one of two processes as described below. Primers modified at the 3’ terminal bases were synthesized using N^6^-benzyldeoxyadenosine and N^6^-tert-butylbenzyldeoxyadenosine loaded Controlled Pore Glass (CPG) substrates to initiate the DNA synthesis. Primers modified at an internal base were synthesized using the N^6^-tert-butylbenzyldeoxyadenosine phosphoramidite. Oligonucleotides were synthesized on an ABI 394 DNA Synthesizer (Applied Biosystems Inc., Foster City, CA) using standard deoxynucleotide phosphoramidites, DCI as activator, and standard synthesis cycles in the conventional 3’ to 5’ orientation on a solid phase CPG support. Following the addition of the last base, the 5’-DMT protecting group was removed on the synthesizer and the oligonucleotide was deprotected (28% aqueous ammonia, 1.0mL, 55ºC, 16 hours). The crude oligonucleotide was evaporated in a stream of air to remove the ammonia, resuspended in water (500µL) filtered through a 0.45 micron syringe filter (GE Healthcare Piscataway, NJ) and purified using Mono-Q HR 16/10 strong anion exchange HPLC column (GE Healthcare,Piscataway, NJ) with a linear gradient of sodium chloride, at pH 12. Fractions were analyzed using a DNAPac PA100 (Dionex Corp., Sunnyvale, CA) ion exchange column and pooled using a minimum purity criterion of >90%. The pooled fractions were desalted by passing the purified oligo solution through NAP-10 (GE Healthcare, Piscataway, NJ) size exclusion columns according to the manufacturer’s directions and evaporated to dryness on a Speedvac (Savant). The primers were formulated to a final concentration of 50 µM in 10 mM Tris, pH 8.0. Purity and yield of the oligo solutions were determined by the DNAPac method above and by measuring the optical density by spectrophotometry. Synthesis of internally-modified primers was carried out using a standard nucleoside loaded CPG and the appropriate modified phosphoramidite synthesized as above.

Synthesis of primers modified with a t-Butyl-benzyl group

Primers modified with p-tert-butylbenzyl groups were prepared analogously from p-tert-butylbenzyl modified nucleoside CPGs exactly as described above.

Oligonucleotide Thermal Melting Curves

Oligonucleotide thermal melting curves (in duplicate) for complementary oligonucleotide pairs at 0.25 μM (A) or 2.5 μM (B) are shown below. The higher temperature melting curves for the first two, longer oligonucleotide pairs from Table 4 are shown in blue (unmodified) and red (modified primer), while the lower temperature melting curves for the last three, shorter oligonucleotide pairs are shown in green (unmodified), black (top strand modified), pink (bottom strand modified), and cyan (both strands modified). The data in Table 4 is derived from the melting curves in figure A with the 0.25 μM oligonucleotide concentration. This data has a noisier baseline than that from the 2.5 μM melting curves, but the lower concentration is similar to that used for primer concentration in PCR. There is a ~4°C shift to higher Tm values for the higher concentration melting curve.

Figure A: Tm experiment at 0.25 micromolar concentrations


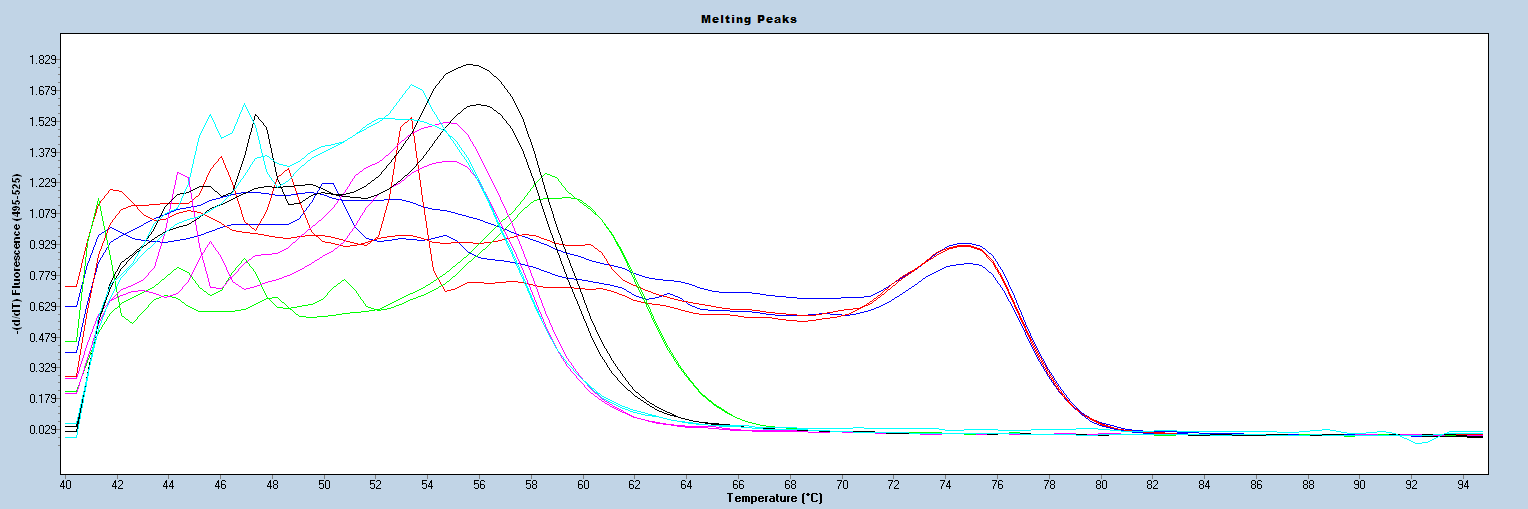


Figure B: Tm experiment at 2.5 micromolar concentrations


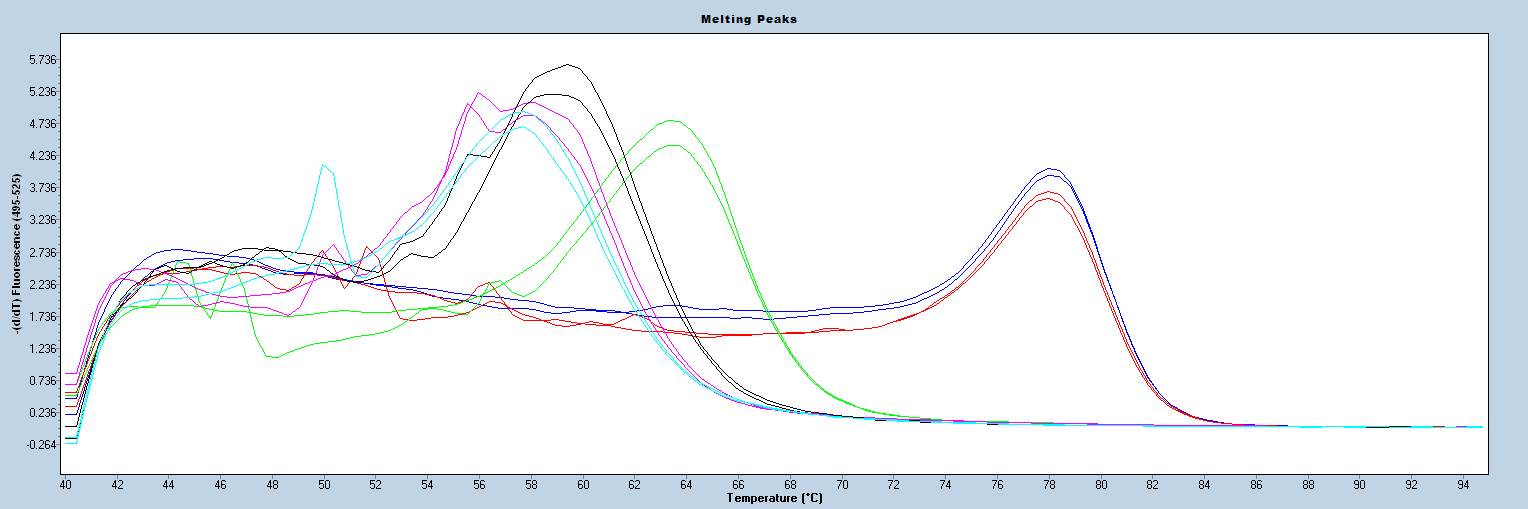


**Abbreviations**

The following standard abbreviations are used

| CPG |  | Controlled Pore Glass |
| --- | --- | --- |
| Ct |  | Cycle threshold |
| DMAP |  | 4-Dimethylaminopyridine |
| DMF |  | N,N-Dimethylformamide |
| DMT |  | 4,4’-Dimethoxytrityl |
| EDC |  | 1-Ethyl-3-(3-dimethylaminopropyl) carbodiimide, hydrochloride |
| LCAA-CPG |  | Long Chain Alkyl Amino controlled pore glass |
| NTC |  | Non Template Control |
| TEA |  | Triethylamine |
| THF |  | Tetrahydrofuran |
